# Qualitative Review of Organizational Responses to Rumors in the 2014–2016 Ebola Virus Disease Outbreak in Liberia and Sierra Leone

**DOI:** 10.9745/GHSP-D-21-00203

**Published:** 2021-09-30

**Authors:** Amelia J. Brandt, Bonnie Katalenich, David W. Seal

**Affiliations:** a Tulane School of Public Health and Tropical Medicine, New Orleans, LA, USA.; b Center for Salud/Health & Opportunities for Latinos, Johns Hopkins University School of Medicine, Baltimore, MD, USA.

## Abstract

**Introduction:**

Rumors and misperceptions were a persistent challenge in the response to the 2014–2016 Ebola Virus Disease (EVD) outbreak in West Africa. This study aimed to document organizational approaches to identifying and addressing rumors and provide practical recommendations for future outbreaks.

**Methods:**

We conducted semistructured interviews with 34 individuals who participated in the EVD response in Liberia and/or Sierra Leone. Interviews focused on the general organizational approach and organizational response to specific rumors. Interviews were recorded and transcribed verbatim.

**Results:**

Most respondents reported that rumors were considered an organizational priority and their importance increased over time. Formal rumor identification systems using community-level reporters were described in Liberia and Sierra Leone as well as varied informal systems. A wide range of approaches was used to address rumors including Community Led Ebola Action, Community Led Total Sanitation, drama performances, Ebola Treatment Center/Unit-based approaches, radio, leveraging community leaders as an information source, and organization change. Interpersonal and community-led approaches were described most often. Staff whose professional roles did not involve rumor management reported informally addressing rumors with colleagues and beneficiaries. Rumors reflecting valid concerns with the EVD response, such as potential infection in health care facilities, were addressed through organizational change and improvement.

**Discussion:**

Interpersonal and community-led approaches were considered effective by participants and hold promise for future outbreaks. Informal systems developed at Ebola Treatment Centers/Units highlighted how these facilities may be utilized as an information hub. Professionals who interact with beneficiaries, especially local staff, are likely to address rumors informally and organizations may benefit from considering local staff an asset in rumor management. Rumors alerted responders to issues in the EVD response, but this may not be the most efficient mechanism to receive and address concerns.

## Abstract (precis)

Rumors and misinformation were a challenge in the 2014–2016 Ebola Virus Disease response and continue to be so in the current COVID-19 pandemic. It is important to understand previous organizational approaches to identifying and addressing rumors to refine and improve these approaches.

## Teaser

**Key Findings:**

- During the 2014–2016 Ebola virus disease outbreak in Liberia and Sierra Leone rumors were identified and managed using formal and informal approaches, most often through interpersonal communication rather than mass media. Rumor management approaches included Community Led Ebola Action, Community Led Total Sanitation, drama performances, Ebola treatment center/unit-based approaches, radio, leveraging community leaders as information sources, and organizational change.
- EVD responders often identified and responded to rumors even when this was not part of their professional role.
- Several rumors were addressed through improvement or changes in the outbreak response.

**Key Implications:**

- Program managers involved in rumor identification and management programming should consider the role of staff members who have direct contact with the public but who do not have specific communication responsibilities.
- Rumors provide vital information about public perception of outbreak response and issues that may require remediation.

## INTRODUCTION

The 2014–2016 Ebola Virus Disease (EVD) outbreak in West Africa was devastating. In Guinea, Liberia, and Sierra Leone there were 28,616 confirmed, probable, and suspected EVD cases and 11,310 deaths.^1^ Although numerous risk and emergency communication manuals, guidance, and trainings existed at the start of the outbreak, persistent communication challenges highlighted gaps in these approaches.^2^ A coordinated response to the outbreak did not begin until approximately 6 months after the first human contact with the Ebola virus^3^ and inadequate and inappropriate communication early in the outbreak proved counterproductive.^4^^–^^6^ Early messaging, for example, overemphasized the importance of bushmeat in transmission while underemphasizing the comparatively higher risk of human-to-human transmission.^7^^,^^8^

Rumors, misperceptions, and community resistance presented significant obstacles in controlling EVD spread.^4^^,^^5^ Rumors, defined as “unverified and instrumentally relevant information statements in circulation that arise in contexts of ambiguity, danger, and potential threat,”^9^ can be problematic, but are an expected and adaptive response to frightening and ambiguous situations.^10^ Information is necessary for planned action and when it is unavailable or not trusted, people may come together to pool information in an attempt to develop a reasonable understanding of a situation.^9^^–^^11^ Rumors are not traditionally viewed as an information source but do reflect fears, hopes, and concerns of the populations in which they circulate. As such, they can be an invaluable resource for informing communication approaches and outbreak response.

This study aimed to capture the range of approaches used to identify and address rumors during the 2014–2016 EVD outbreak in Liberia and Sierra Leone. Guinea was excluded because the interview guide drew on rumors previously identified in Liberia and Sierra Leone, and rumor data were not available for Guinea.

Although program descriptions describing formal organizational responses to rumors exist,^4^^,^^5^^,^^12^^,^^13^ this study sought to describe both informal and formal approaches used by EVD responders in a variety of roles, locations, and organizations to identify and address rumors to provide practical recommendations for rumor identification and management in future outbreaks. Rumors continue to be of interest in public health as illustrated by their impact on the ongoing COVID-19 pandemic.^14^^,^^15^ To better understand how to identify and manage rumors, it is important to identify lessons learned from previous outbreaks and encourage innovation. In particular, studying informal rumor identification and management techniques practiced by EVD responders with direct community contact can provide unique insights into bottom-up approaches to identifying and addressing rumors.

Studying informal rumor identification and management techniques can provide unique insights into bottom-up approaches to addressing rumors.

## METHODS

This was a cross-sectional qualitative study.

The study was reviewed and approved by the Tulane University Social-Behavioral Institutional Review Board. Participants provided oral informed consent.

### Sample

Participants were recruited via Facebook and Twitter posts on the lead author’s personal pages, the lead author’s professional networks, and the CORE Group, Global Alliance for Nursing and Midwifery, and Healthcare Information for All listservs. Snowball sampling was used to reach the target sample size of 30 to 50 or until saturation was reached.

Thirty-four individuals who participated in the EVD response in a professional capacity in Liberia or Sierra Leone participated in the study. Of these, 16 participants worked in Sierra Leone, 14 worked in Liberia, and 4 worked in both countries. Twenty participants were international staff and 14 were local staff. Individuals aged younger than 18 years and/or unable to communicate in English were excluded.

### Data Collection

First, participants completed a short survey about the organizations they worked with during the EVD response. The survey was available on the Health Insurance Portability and Accountability Act (HIPAA)-compliant Johns Hopkins University instance of Qualtrics.

Then, participants completed semistructured interviews on organizational response to common rumors about EVD. Interviews were conducted in person, by phone, or via their preferred digital platform (e.g., Skype, WhatsApp, Zoom). Interviews conducted via Skype and Zoom were audio-recorded using platforms’ integrated recording capacity. All other interviews were recorded via QuickTime Player version 10.5 on a laptop computer. Audio recordings were labeled with a participant code and transcribed verbatim.

Data collection took place between September 24, 2019, and June 24, 2020.

### Data Analysis

Survey data were downloaded from Qualtrics and analyzed in Microsoft Excel. Interview transcripts were analyzed in Dedoose version 8.3.21 using individual-level thematic content analysis based upon an a priori analysis plan (Table 1). Two coders reviewed the first 20 interview transcripts to develop a codebook based on emerging themes. Each transcript was coded by 1 coder and reviewed by a second coder who identified code discordance. Coders discussed discordant codes until reaching consensus. Participants were not provided with a rumor definition but were rather asked how they defined rumors. This was an intentional approach to allow participants to describe their experience based on their own understanding of rumors and how they were addressed.

**TABLE 1.** Analysis Plan for Semistructured Interview Responses of Participants Who Were Asked About Their Organization’s Response to Rumors During the 2014–2016 Ebola Virus Disease Outbreak

**Interview Topics**	**Analytic Questions**
Ebola virus disease responder background and experience	MICRO^1^: What is the background of this participant? MICRO: What perspective does this participant have regarding rumors? MACRO^2^: How does this participant’s background correspond to her perception of rumors?
General organizational approach to rumors	MACRO: What is the range of organizational approaches to rumors?
Organizational response to specific rumors	MACRO: What is the range of organizational responses to specific rumors?

^1^ Refers to analytic questions that focus on the individual participant.

^2^ Refers to analytic questions that focus on trends amongst all participants.

## RESULTS

### Participant Background and Rumor Perception

Of participants who completed the organizational characteristics survey (n=30), 17 worked for 1 organization during the outbreak, 11 worked for 2 organizations, and 2 worked for 3 or more organizations. In total, participants reported working at 40 organizations (Table 2).

**TABLE 2.** Characteristics of Organizations Employing Interview Participants Who Were Asked About Their Organization’s Response to Rumors During the 2014–2016 Ebola Virus Disease Outbreak, by Country

**Organizational Characteristic**	**Liberia**	**Sierra Leone **
Organization type	n=17	n=23
Bilateral cooperation organization	1	0
Governmental organization	1	3
International nongovernmental organization	11	18
Local nongovernmental organization	0	2
United Nations agency	1	0
Other	3	0
Number of in-country employees	n=17	n=23
<10	2	1
10–25	2	2
26–50	5	2
51–100	3	3
101–500	5	5
>500	0	9
Don't know/not sure	0	1
Number of total employees	n=13	n=20
26–50	0	2
51–100	3	0
101–500	1	0
>500	8	10
Don't know/not sure	1	8
Not applicable	0	0
National headquarters location	n=13	n=20
Capital city	11	17
Other	2	3
Participation in national coordination structure^a^	n=17	n=23
Case management	6	19
Epidemiological/surveillance	6	14
Laboratory	2	9
Social mobilization	9	21
Contact tracing	5	N/A^b^
Special staff	4	N/A^b^
Logistics	N/A^b^	11
Burials	N/A^b^	7
Psychosocial	N/A^b^	18
Other	8	7
Don't know/not sure	1	0

^a^ Organizations participated in multiple coordination structures.

^b^ Organization structure did not exist in this country.

The professional and educational backgrounds of participants varied and included international development, emergency response, public health, and clinical care, with international development and public health being the most common. Many participants had experience in multiple areas.

### Organizational Response to Rumors

#### Rumor Priority

At an organizational level, the importance of identifying and addressing rumors varied. Some participants reported a high-priority level but also described how the priority level varied between departments. Several participants indicated that rumors were a low priority in their organizations. Increasing priority of rumors over time emerged as a common theme. Table 3 presents illustrative quotes regarding rumor priority.

**TABLE 3.** Participant Quotes Regarding Rumor Priority From Interviews on Organizational Response to Rumors During the 2014–2016 Ebola Virus Disease Outbreak in Liberia and Sierra Leone

**Theme**	**Quote**
High priority	Rumors were super important, and they were considered a fundamental bedrock of everything that they were doing. —International staff, Sierra Leone
Priority variation within organization	I think we really dropped the ball at the [Ebola Treatment Center]. I mean, it's embarrassing because … it should have been really at the top of my mind, but it wasn't. I was so busy doing other ETC stuff. I wasn't thinking about laying any kind of groundwork or trying to track rumors around the ETC. —International staff, Sierra Leone
Low priority	As far as I remember - they were not particularly concerned in having an approach to do with addressing the rumors. —International staff, Sierra Leone
Changing priority over time	I don't think anyone really knew what a huge problem they would be until after the peak of the outbreak. I don't I don't think we understood how much of a barrier they would be. I mean we started hearing rumors in probably May or June of that of 2014, but I don't I just don't think any of us foresaw what a barrier they would be to health seeking behaviors. And so before then I don't think we took them seriously, to be quite honest. Or not seriously enough. I think there was a lot of, like, laughing them off at the Ministry level and in the technical working groups. —International staff, Liberia

#### Rumor Identification

Of the 34 participants, 13 described a formal rumor identification system. Formal systems described in Liberia and Sierra Leone consisted of a national network of community-level individuals reporting rumors via mobile phone. Additionally, a few participants also mentioned formal systems reviewing mass media to identify rumors. However, although the formal systems were national, several participants were unaware of their existence.

Formal rumor identification systems in Liberia and Sierra Leone used a national network of community-level individuals reporting rumors.

Many participants discussed how organizations identified rumors with informal or ad hoc methods, most often through in-person communication between staff and community members. In some cases, staff members who were told about rumors worked in social mobilization or communication roles, although in some cases local staff were asked about rumors even if their professional role did not include any formal communication responsibilities. For example, a local staff working with an international nongovernmental organization focused on creating business opportunities described how he was frequently asked about rumors. Some participants also described other systems used to identify rumors of EVD cases but not other types of rumors. Table 4 presents illustrative quotes regarding the rumor identification processes.

**TABLE 4.** Participant Quotes Regarding Rumor Identification From Interviews on Organizational Response to Rumors During the 2014–2016 Ebola Virus Disease Outbreak in Liberia and Sierra Leone

**Theme**	**Quotation**
Formal System – Community Level Worker Network	You know, we had a network of … over 10,000 people. If you put the religious leaders, the social mobilizers, the traditional healers together that we are working with … With the social mobilizers, we had we developed a system of communication. … On a daily basis the mobilizers were able to share … rumors. The things we hear from the communities as they [were] engaging the community … So we are getting those. I will not say close to real time, but on a daily basis we are able to actually collect that information. —Local staff, Sierra Leone
	We would work with Community Health volunteers who would send via [short messaging system] the specific rumor that they heard and that would track the [global positioning system] coordinates. And so then what would happen is we could say ‘okay, this 1 specific rumor came from this specific village on this specific day,’ and then we could alert our social mobilizers as well as the Ministry contacts that we had about that specific rumor. —International staff, Liberia
Formal System – Mass Media	That was a separate pillar that was led that was co-led by the Ministry of Information and they really … were more responsible for gathering some of that misinformation that was spreading … through the media, through … some of those channels. And so both that pillar, and the social mobilization pillar would bring that information to those daily Situation Room meetings where it was discussed then with the wider … response teams. —International staff, Liberia and Sierra Leone
Informal System	I mean it was it was an everyday thing where … I mean most of the time when I would do the work, people would confront us and talk to us about these rumors. And, you know. But we knew exactly what it is. And then we knew where the rumors came from. —Local staff, Liberia
System to Identify Ebola Virus Disease Case Rumors	The rumors … [we] will get the call from community-level. At times they too come from stakeholders [and] at times it will come from our partners that were working with the [nongovernmental organizations]. They will bring the information to us and then we would investigate the rumors and then if it is true then we do investigation and then if necessary, we can collect samples. —Local staff, Sierra Leone
	[It] …was like, ‘Oh if you hear that there is Ebola, you need to report to authorities.’ There was even a toll-free line where you could call … so that a response team could come and address to that case. —International staff, Sierra Leone

### Rumor Management

Participants described a wide range of rumor management approaches using interpersonal communication and mass media channels. Participants emphasized that using multiple communication channels was vital to ensure that people in remote or isolated areas were reached, as well as the importance of different complementary approaches (e.g., radio, community meetings, drama) that allowed for wide distribution of information as well as gave people opportunities to ask questions and raise concerns.

Interpersonal communication approaches were discussed most often and were generally implemented at the community level. In Sierra Leone, one such approach was a participatory methodology called Community-Led Ebola Action. One participant described how this approach was used to address fears and rumors about personal protective equipment and ambulances. Participants described a similar approach that was used country-wide and coordinated through a consortium. Another international staff member in Liberia discussed how Community-Led Total Sanitation, the approach on which Community-Led Ebola Action was based, was adapted for community engagement in Lofa County, Liberia. Community-Led Ebola Action^16^ and Community-Led Total Sanitation^17^^,^^18^ have previously been described in detail in the literature. These approaches emphasize the capacity of communities to develop their own solutions to challenges that meet scientific standards for outbreak control as well as the importance of demystifying situations and objects that cause fear. Another community engagement approach used musical and drama performances. Participants also discussed the importance of listening to questions and concerns from the community, gathering accurate information, and sharing that information with the community to close the communication loop.

Community-level communication approaches emphasize communities’ capacity to develop solutions to challenges that meet scientific standards for outbreak control and help demystify situations and objects that cause fear.

Informal interpersonal communication efforts were also frequently discussed, especially those that took place in the Ebola treatment center/unit (ETC/ETU) context. Another international staff member working at an ETC/ETU described a more formal approach to using downtime to provide education and reduce misinformation. Although this approach used interpersonal communication to start, it later expanded to mass media. These approaches illustrated the potential for the ETC/ETU to be a setting for information sharing and addressing rumors.

Radio was the most common medium for mass media approaches, but billboards, posters, and social media were also mentioned. Interactive radio programs were generally seen to be the most effective mass media efforts. One international staff member working in Liberia and Sierra Leone and described other radio programming and efforts to work with journalists to reduce and address rumors.

Consistent messaging was discussed as a key attribute to both mass media and interpersonal communication approaches. In Sierra Leone, participants described a standard messaging guide that was produced by a Social Mobilization Action Consortium, while in Liberia participants reported that official messaging was centrally approved. Although consistent messaging was considered important to effective communication, several participants also discussed how initial messaging about the high likelihood of death from EVD was actually counterproductive and caused people to hide EVD cases. This illustrated the importance of considering the potential unintended effects of messaging.

A common theme in participants’ discussions of both interpersonal and mass media rumor management approaches was the importance of delivering information through trusted sources. The first step described for identifying trusted sources in communities was entering a community in a culturally appropriate way. One international staff member working in Sierra Leone discussed the need to be cautious in entering communities; approaches that worked in some communities may not work in others. After entering a community, participants discussed approaches to identifying trusted community members and then using those people to share information as community members may be more likely to accept information from a trusted source.

While the majority of rumor management approaches discussed were focused on communication, several participants also provided examples of organizational changes. For example, several participants discussed how family access to the ETC/ETU was increased over time and decreased rumors. Some organizational changes were made in direct response to a rumor. For example, bags used for safe burials were modified to allow family and friends to see the face of the deceased in response to rumors that these bags were filled with rocks, which illustrated the importance of transparency in reducing rumors.

Several participants discussed how traditional beliefs could affect communication and rumor management. One international staff member working in Sierra Leone described how this appeared to contribute to the persistence of the rumor that EVD was not real. In contrast, a local staff member also working in Sierra Leone described how they took traditional beliefs into account to lead to desired health behaviors. This contrasting approach illustrated that traditional beliefs, even those that are inconsistent with the biomedical explanation of disease, may not be inconsistent with behavior change goals.

Another theme that emerged in several interviews was how to address rumors that the participants felt were based on truth, at least in part. Participants mentioned rumors of people profiting from EVD, chlorine being dangerous, nepotism in ETC/ETU hiring, and health workers transmitting EVD as having some basis in truth. Participants generally indicated that when addressing these rumors the best approach was to acknowledge the element of truth. Table 5 presents interview quotations for emergent rumor management themes and summarizes the key insights for rumor management approaches.

**TABLE 5.** Participant Quotes and Key Insights Regarding Rumor Management From Interviews on Organizational Response to Rumors During the 2014–2016 Ebola Virus Disease Outbreak in Liberia and Sierra Leone

**Theme**	**Quotation**	**Key Insights**
Use of multiple approaches and channels	I think what is important is having a combination of all these channels so that you’re sure you're reaching people even the remotest areas of the country. So although you know committee meetings are better because you can have conversations, people can ask questions, and you respond but in terms of creating awareness and making sure you reach as many people as possible, I think a combination of all these channels was quite important. Because there’s also posters that are put out there, so for those that way are able to read in order to get that information and based on what they've seen on posters, for example, they, during committee meetings, they could go ask more questions or based on what they would have heard on the radio, when they come face-to-face with a health worker, they have an opportunity to ask questions. —International staff, Sierra Leone	Importance of using multiple complementary channels to disseminate information
Community Led Ebola Action	You have the community champions. You have those to follow up so they were also used because the mobilizers during those triggering were able to listen to the communities to get the community perspective about how they could also protect themselves and keep themselves safe because honestly speaking I think most times we think the communities do not have … a scientific explanation to how they are able to prevent or protect themselves against diseases like Ebola and that. But if you engage the communities, really and you sit with them you discuss then you realize that they also have some explanations and some ideas on how they would be able to do it and that meets scientific standards. —Local staff, Sierra Leone	Communities are capable of developing their own solutions that meet scientific standards
	People … were resisting because of the PPE (personal protective equipment people are putting on. … During the community engagement we had a session where you have community [members] who were actually encouraged to wear PPE …. You have Community leaders all using the ambulance and … so people were going to the ambulance so that the community that we serve will see that's okay. ‘Okay, my fear was able to actually go into the ambulance and nothing happened.’ So it means though that was able to kind of demystify some of the myths around the use of ambulance and the PPE that people were afraid that were leading to some of … [that] resistance. —Local staff, Sierra Leone	Demystifying situations or objects that cause fear can help reduce rumors.
Community Led Total Sanitation Adaptation	[It] was a like a way of bringing community members together in groups and addressing like their fear and their perceptions and their experiences and stories they'd heard and all those kind of like listening to all of that and then giving them the actual information and kind of addressing directly those, you know, maybe misconceptions that they had or clarifying if they were correct ones and then helping them to figure out what they need to do. —International staff, Liberia	It is important to listen to community questions and feedback, gather appropriate information, and communicate back with the community to close the loop.
Drama performance	We didn't just go and perform but we created a stage that will reduce the number of rumor because people ask questions that they feel somebody told them, and we wrote those questions down and we communicate[d] their back with our sponsor, like UNICEF, right … and they will send the real information. —Local staff, Liberia	
Ebola Treatment Center/Unit-based approaches	But I suppose the nice thing was that we had really great relationships with the Ebola Treatment Center staff and they were there, you know every day, so you had time to like just sit and shoot the sh** with them and if they were like ‘this is what I'm hearing,’ then you had time and space to just be like, ‘okay, let's talk about why the, you know, physiology of Ebola isn't actually related to, you know, kind of airborne spread like, so let's kind of talk about that. So kind of trying to defeat those rumors on a very one-to-one personal kind of basis. —International staff, Sierra Leone	The Ebola Treatment Center / Ebola Treatment Unit was a useful setting for information sharing and addressing rumors.
	We started this whole training curriculum where you know, they [local staff] … would take responsibility and sign up for topics and then go research it and then teach their peers about that particular topic, and then we included the foreign staff and stuff, and, and tracked it. So we treated it like, almost like an education degree that was informal. … The nursing team also started a weekly radio show where they had like call in questions … The local … Ebola Treatment Unit team, that was a way that they wanted to be available to answer questions. It was so cool. They did, I remember, they did … topics at different times, like one was about fever, and then they would talk about Tylenol, and talk about what a fever is doing for someone's body, and it was linked to [the] … curriculum that we set up with our foreign nursing staff. —International staff, Liberia	
Radio	I think in terms of addressing rumors, the best way was usually through interactive programs. So whether you have an interactive program on radio, TV, or at a community level, programs where people can ask questions and answers are provided. I think those were the best in terms of addressing rumors, because sometimes the rumor is spread about a particular issue and then you respond with a messaging without getting to hear from the people who are spreading the rumors, but I think the best ones always platforms that give people an opportunity to ask questions and get responses. —International staff, Sierra Leone	Interactive radio programs were perceived to be the most effective.
	Radio dramas that were that were done at that time trying to remember the name of it. Mr. Plan-Plan or something like that, but there were you know some radio dramas and radio programming that were that were broadcast that would touch on addressing some of those some of those rumors. There were the journalist trainings. So to make sure that, you know, journalists were reporting more accurately. So there are a couple other things that that were being done at that time as well that, you know, would help to provide more accurate information, which would hopefully then reduce the number of rumors … And you know, maybe one thing to add is that you know, I think there was a real sense of wanting to be very careful about not repeating the rumor. So, you know by addressing the rumor you're really kind of just accelerating that accurate information rather than repeating the rumor in a way that's you're telling people that it's not true. So that's something that you know where people were really careful not to do. Because I didn't want to exacerbate that that rumor. —International staff, Sierra Leone and Liberia	Journalist training can help prevent rumors by increasing accuracy of media reports.
Centrally approved messaging	It was the kind of feeding up from the ground and then feeding back down. Kind of changing up the message guides like every couple of weeks or every month to ensure that they had. That they were kind of addressing the most current rumors. —International staff, Sierra Leone	Messaging was centrally approved in Liberia and Sierra Leone.
	So if the information came from Central Ministry because Central Ministry is responsible to do the IEC/BCC (information education communication/behavior change communication) had been approved. So if that was done, it came down to the county, from the county to the district, and then to your community that needed the information. —Local staff, Liberia	
Ineffective messaging	But at first, it was just like, it kill[s] you, you will die. It will kill you. But later on now the message change[d] … On the message side we change that, look, when you have this, you can get to the treatment center. You'll be treated and you can survive. People became relaxed. But during the early stages a lot of people ran away. —Local staff, Liberia	It is important to consider potential unintended effects of messaging.
Community entry strategies	If you just show up with you know and have, you know, PPEs you know with chlorine sprayers and start giving messages, if you're lucky, the best thing … is they won't listen to you. The other thing is … run you out of the village. —International staff, Liberia	Community entry is a sensitive process that needs to be done carefully and in a culturally appropriate way.
	If you go to a village that you know is infested, you call the Elder, like the Big Chief. Have him call his people because there were no mobile phones in order to make an appointment … Having called the elders, called the people, and tell them look we're coming back the next day because we're doing it from town to town. Like we spend a whole week out of town because you actually make appointments. Like, look we coming back tomorrow, Saturday at 4 o'clock. Let other people be here. We got a bundle of good news for you. —Local staff, Liberia	
	In some of the chiefdoms that we worked in, the paramount chiefs were very well respected, and they were sort of the purveyors of a lot of trust, and a lot of trying to you know, if you wanted access to the community you had to go through them. I know that there were some communities that we worked in or that we worked with that was the opposite, is that the Paramount Chief was not seen as being legitimate. It was somebody who was put in place because they were, you know, somebody's father or brother or connected somebody who is already powerful, and so the community actually didn't trust them. —International staff, Sierra Leone	Entry strategies vary, so it is important to gain an understanding of the community leadership structure before entering.
Community leaders as information source	You have to go you have to drill down deeper and find out who in each community is already an accepted source of information and whether they are knowledgeable about the issue, whether it's Ebola or an earthquake or whatever … All communities have some natural leaders. Some of them are either formally appointed like the village Chief or Council of some kind or you have just individuals who are recognized by other members of the community as having skills of organizing people of putting together trainings or those kind of things. —International staff, Liberia	Communities may be more likely to accept information from a trusted source in the community rather than from an outsider.
	And if there is any new information you share with people … so that it comes from the trusted source first. Otherwise, if you try to counter it, it's difficult. With misinformation, you just continue to provide accurate information. Probably through trusted channels in the community. So like religious leaders, traditional leaders, and mobilizers who are residing in the communities … You may be able to use those people to provide information to the populace regarding rumors. —Local staff, Sierra Leone	
Organizational change	One of the things that was changed in the short time I was there, was the opening the possibility for the family to see the people inside the treatment center, with quite a distance but being able to communicate with them. And the other thing that was, I think, quite important was also … opening the possibility in the treatment center, to see the dead body from the distance. —International staff, Liberia and Sierra Leone	Increasing family’s ability to communicate and see their loved ones in the Ebola Treatment Center / Ebola Treatment Unit increased trust and reduced rumors.
	I do think actually the changes that were made at the treatment centers to increase a patient's ability, family's ability to see the patients I think that that has a huge impact, on trust and rumors, I think in a lot of ways probably more so than communication. —International staff, Liberia	
	The burial team … [was] hearing rumors about how you know, the body bags that they were using were, you know, the bodies weren't in it. They were taking the bodies out. I think again there were rumors around harvesting organs and other things. And that … that they were filling those bags up with rocks. And so what [organization] did was they … [made] part of the body bags … a see-through screen basically so that you could at least see the face of the body. —International staff, Liberia and Sierra Leone	Increasing transparency, where possible, may reduce rumors.
Traditional beliefs	Strong support of traditional beliefs and the fact that there was a really strong tradition in the spirit world allowed for alternative explanations for what was happening [that] did not involve germ theory. And trying to convince a population that doesn't have a high scientific background, that viruses exist and that this is how you pass the virus from one person to another. And … just not realizing viruses exist but that they can kill people, and that your traditional, really important practices of your community like making sure people are buried properly and taking care of loved ones in the home, are putting you and your family at risk too. I think that the biggest issue with the sustained rumor was lack of scientific education combined with strong traditional beliefs that provided a really solid counter-narrative to what was going on. —International staff, Sierra Leone	Although traditional beliefs may lead to a rejection of the biomedical explanation of disease, it is possible to accept these beliefs and incorporate them into behavior change communication.
	We have a discussion with stakeholders. And then we explain to them that what they are calling voodoo, medically we call it an illness. It can be carried by illness. So we educate them. At times we tell them that, ‘okay, the names are different.’ We tell them for example, if we're talking to religious leaders, we tell them … ‘there are many names people call God. So that's the same medically there are many ways what you call witchcraft is what we call a sickness. So this sickness we need to … investigate it and tell you what the exact sickness is and then we can give medication and the person will be okay.’ For example, when we had the outbreak here in my own community, they said … a witchcraft plane had a crashed. So as a result many people were dying. So we accepted the fact. We told them ‘okay, yes what you call a plane crash is what we call medically Ebola.’ So it's like we buy the idea so that they can accept us and there we have a deal where we explain to them. We give them the right information. That's when we will have an agreement and then they will listen to us and then we work as a team because [for] community infection you need community intervention so that at the end of the day you are able to achieve your aims. —Local staff, Sierra Leone	
Addressing rumors with element of truth	I would default to saying that it's unhelpful to reject or disagree with those narratives that people are making money in the response because it is true. And if they're rejected, if the response given to those individuals is, "No, no, like, you know, it's nothing to do with that. That's not why we're here and you're wrong," then I think it makes it a lot more difficult to convey and convince them of other … It doesn't present yourself as a trustworthy source of information. I suppose is the best way to put it. —International staff, Sierra Leone	When rumors have an element of truth, it is best to acknowledge it.
	I mean, chlorine's a corrosive gas, like it's real … and they used it … And in fact the new guidelines for spraying of chlorine, do not include spraying because aerosolized chlorine (A) does not actually kill the virus, and (B) it gets it into your lungs and it can cause some pretty harmful effects on the lungs, especially for people who've been working in treatment centers or on decontamination teams, whatever and they're constantly inhaling the sh** … A lot of people would say ‘oh, we don't like the chlorine’ and I'd be like, ‘well, this is the protocol.’ And I think the logic that I had at the time was you're more likely to die of Ebola than you are chlorine poisoning. Or like, you know, it's a long-term impact. What we're trying to do is stop you from dying today. —International staff, Sierra Leone	

### Organizational Response to Specific Rumors

A common rumor in Sierra Leone was that health workers were spreading EVD. Participants mentioned identifying the rumor in several different ways.

A common rumor in Sierra Leone was that health workers were spreading EVD.

Five of the 9 participants who discussed this rumor believed that there was some truth to this rumor, but several believed the fears may have been overstated. Despite participants believing that there was some element of truth to the rumor, they also recognized that it was very damaging and led to the stigmatization of health workers and decreased health-seeking behavior.

The primary approach to addressing this rumor was organizational change and improvement of infection prevention and control procedures. As 1 participant put it:

*You can't message your way out of something like that.* —International staff, Sierra Leone

Interpersonal communication approaches and mass media campaigns were also used to complement organization change efforts. Table 6 presents illustrative quotes describing the organizational response to this rumor.

**TABLE 6.** Participant Quotations Regarding Organizational Response to Rumors of Health Workers Spreading Ebola Virus Disease in Sierra Leone

**Theme**	**Quotation**
Rumor identification	I remember hearing it even before I was in Sierra Leone. I remember reading news stories. —International staff, Sierra Leone
	It was during the Monday morning meetings when I went to Kenema. So the SMAC team who was talking about this during the Monday morning meetings. —International staff, Sierra Leone
	Working in surveillance, I just remember seeing that there were some communities that were reluctant to report and I started asking the community members when I would go out for a certain case investigations with a surveillance team and then ask the surveillance team why they weren't speaking, why they weren't reporting, and they would tell me it was 1 of those 2 reasons, [that health care workers spread EVD or chlorine is deadly]. —International staff, Sierra Leone
Element of truth in rumor	I don't think that there's an impossibility that people who went into an Ebola Treatment Center and were negative didn't pick up anything while they were in the Ebola Treatment Center and then get sick because they were there. I do think as well that there is a truth to the fact that some health care workers were treating patients at home and not necessarily in full [infection prevention and control] procedures and because they were treating people at home then they themselves might have gotten sick and could have potentially then passed it on to other people if they were treating them as well … I think that it's not that there was no truth to the fact that health care workers can spread Ebola. I think that the rumor that health care workers were spreading Ebola to the extent that that was being feared was probably not true. If somebody was treating somebody and got sick and then continued to treat people while they were themselves sick, yes, they could absolutely have spread Ebola, but usually when people get sick, then they're too sick to continue being clinicians and not always, obviously, but most of the time I think that people are not necessarily able to provide medical care at that point. —International staff, Sierra Leone
Negative effects of rumor	During the time of Ebola, people were afraid to go to the hospital, people were afraid to go to the health centers. And so in Sierra Leone health workers were victims. So health workers were kicked out of their places that they are living … Landlords gave notice to health workers ‘we want you no more, you are kicked out of your home Because if you are a health worker, you might have Ebola, and you might infect us.” —Local staff, Sierra Leone
Rumor management: organizational change	You can't do an SBC campaign until you actually fix the [infection prevention and control] problem. So a big part of it is you've actually got to just make the [infection prevention and control] better at the level of the health facility and then you can start to bring people in and say let's go for a tour of this facility. Why don't you participate in an evaluation of the infection prevention and control of the facility, use the rumor phone call line to let us know if people are not wearing gloves or if they're reusing needles, you know, ask people to sort of be agents like have some kind of they have to be agents of making it better I think a lot of the time. It's about working with health care workers to understand that [infection prevention and control] is not an optional but it's also a way of keeping them safe from sickness. I think you've really got to attack it on both supply and demand side if you want to get some measure of trust back into health system or in a Health service. —International staff, Sierra Leone
	I think we just got to make a better … response. Right? I think one way to beat those types of rumors … is to put up results that show that essentially protect people and doesn't exacerbate the issues that we're all talking about. So I feel like that's really the only way to do that, the only way to really truly build trust after trust has been broken through a number of different things where it's rumors that have truth but are damaging nonetheless would be to build trust through proving that we can do this right. Which is can we reduce health care worker infections and nosocomial infections? When persons that are believed to be sick interact with health care workers and response workers as well. —International staff, Sierra Leone
Rumor management: interpersonal communication	We hire people from the communities educated about … Ebola, and they go out and talk to their people telling - giving them the right information about the virus, right information about how to … and so that you keep yourself safe. So we are doing the IEC: information, education, and communication, through them. Where we mobilize communities, we explain to them about tell them about the rumors that people are talking about because of communities first talk about the rumors, what they are hearing … we asked them about their information on the virus, on the outbreak, and they tell us what they know, where they had got the idea in from other people, and on what they are saying. We give them the right information, we give them posters, we give them flyers, we show them videos that we would use about the outbreak. And we would get most of the people convinced that the rumors that they were hearing were not…. So this was one of the way that we are tackling about rumors. —Local staff, Sierra Leone
Rumor management: mass media	They tried to hold up people who were survivors and people who were Ebola champions to show what their contribution had been in terms of fighting Ebola and the fact that basically that they were to be trusted and they highlighted a lot of the Ebola response workers and the health care workers in that, which was really cool. —International staff, Sierra Leone

## DISCUSSION

Rumor identification systems using mobile phones and large networks of social mobilizers appeared to be effective for gathering rumors, but interviews seemed to indicate that there was limited awareness of these systems. This indicates a need for improved coordination and communication about rumor identification and management efforts. It is also worth considering how information from rumor identification systems can be used to improve outbreak response. While rumors and misinformation are often considered within the purview of communications, rumors are also an important information source regarding how operations can be improved. This was illustrated by the operational changes that several organizations made in response to rumors in the community.

It also was a consistent theme that EVD responders heard and responded to rumors even if this was not part of their professional role. This was especially common among local staff. This presents a potential risk as staff members may respond in a way that exacerbates a negative rumor but also presents an opportunity. Organizations can use their internal communication structures to encourage staff members to report these rumors and provide the necessary information and skills to respond to them. For example, it could be beneficial to allocate a portion of time during staff meetings to discuss questions, concerns, and rumors that staff are hearing from the communities they work with. Alternatively, an organization could appoint a focal person to which that community feedback could be directed. Social and behavior change training (effective communication, facilitation, negotiated behavior change, etc.) could be beneficial to prepare staff members to respond to rumors. These efforts should take into account existing workloads and be cognizant of situations where this would cause an undue burden on staff members.

Organizations can use their internal communication structures to encourage staff members to report rumors and provide the necessary information and skills to respond to them.

The use of mass media messaging and interpersonal communication at a community level to address rumors is not a new idea. Many EVD responders discussed how the 2014–2016 EVD outbreak was a learning experience and that both mass media and interpersonal communication approaches steadily improved as the outbreak went on. However, this type of approach may underutilize rumors as an information source. The results of this study illustrate how rumors highlighted issues within the response that were later addressed, but this process was ad hoc as there was not a structure in place for community members to share rumors or feedback directly with those who could address their concerns.

Despite this challenge, numerous examples of how community feedback was used to improve the outbreak response were described. One of the most striking examples was the rumor that health workers were spreading EVD. This rumor was perceived by several EVD responders as having some basis in truth and is supported by evidence of nosocomial transmission during the outbreak in Sierra Leone.^19^^,^^20^ As such, the primary response to this rumor was no communication or messaging, but rather improving infection prevention and control procedures in ETC/ETUs and health centers. Rumors that indicated mistrust of ETC/ETUs were seen by most EVD responders as being understandable given that in the early part of the outbreak family members or friends often went to the ETC/ETU and were never seen again. Increasing access to ETC/ETUs and modifying burial practices to allow safe family participation was perceived by participants to successfully reduce rumors, but more importantly, led to a more compassionate response that respected the humanity of those affected by the outbreak.

### Limitations

This study had several limitations. EVD responders working with large international nongovernmental organizations were overrepresented in the sample due to the use of snowball sampling and the author’s own professional network. The lead author had worked in Sierra Leone during the 2014–2016 EVD outbreak, which resulted in higher levels of interconnection between participants who had worked in Sierra Leone and may have contributed to more similar opinions among those participants. No incentive was provided for participating in the study so it is likely that participants were motivated primarily in their own interest in the topic, which may have led to some sampling bias. To encourage openness and honesty in participant interviews, participants were not asked to provide the name of the organization(s) they worked with. However, as organization names are not included in the data, it is not possible to take into account duplication in the organizations represented. The data were also limited due to the long amount of time that had passed between the EVD outbreak and the time of interviews.

## CONCLUSION

This study provides an overview of how rumors affected the 2014–2016 EVD response at an organizational level. While participants believed rumors had a clearly negative impact on health-seeking behavior, they were also instrumental in improving the EVD response. This study illustrates that rumors should be a key consideration in outbreak responses from the start of an outbreak response and should be considered in all aspects of the response, not only as an issue to be addressed via communication.

## Notes

**Peer Reviewed**

**First published online:** August 17, 2021.
